# The Role of the PRMT5–SND1 Axis in Hepatocellular Carcinoma

**DOI:** 10.3390/epigenomes5010002

**Published:** 2021-01-05

**Authors:** Tanner Wright, Yalong Wang, Mark T. Bedford

**Affiliations:** 1 Department of Epigenetics and Molecular Carcinogenesis, The University of Texas MD Anderson Cancer Center, Smithville, TX 78957, USA; TWright3@mdanderson.org (T.W.); YWang68@mdanderson.org (Y.W.); 2 Graduate Program in Genetics & Epigenetics, UTHealth Graduate School of Biomedical Sciences, The University of Texas MD Anderson Cancer Center, Houston, TX 77030, USA

**Keywords:** arginine methylation, PRMT5, SND1, Tudor-SN, p100, HCC

## Abstract

Arginine methylation is an essential post-translational modification (PTM) deposited by protein arginine methyltransferases (PRMTs) and recognized by Tudor domain-containing proteins. Of the nine mammalian PRMTs, PRMT5 is the primary enzyme responsible for the deposition of symmetric arginine methylation marks in cells. The staphylococcal nuclease and Tudor domain-containing 1 (SND1) effector protein is a key reader of the marks deposited by PRMT5. Both PRMT5 and SND1 are broadly expressed and their deregulation is reported to be associated with a range of disease phenotypes, including cancer. Hepatocellular carcinoma (HCC) is an example of a cancer type that often displays elevated PRMT5 and SND1 levels, and there is evidence that hyperactivation of this axis is oncogenic. Importantly, this pathway can be tempered with small-molecule inhibitors that target PRMT5, offering a therapeutic node for cancer, such as HCC, that display high PRMT5–SND1 axis activity. Here we summarize the known activities of this writer–reader pair, with a focus on their biological roles in HCC. This will help establish a foundation for treating HCC with PRMT5 inhibitors and also identify potential biomarkers that could predict sensitivity to this type of therapy.

## 1. Introduction

Signal transduction is the process by which information is relayed through a cell. Extracellular signals, such as growth factors or contact points with other cells, stimulate receptors on the cell surface to initiate this process by converting one stimulus (ligand binding) into another (phosphorylation). This signal initiation event is then propelled through the cytoplasm and into the nucleus using a series of sequential PTM events that rely on “reader” proteins, or effector molecules, to dock onto a specific PTM and then promote the deposition of a new PTM downstream, which in turn is read and relayed by another effector. There is a vast array of different PTMs including, but not limited to, phosphorylation, acetylation, ubiquitination and methylation, which can all be read by specialized protein domains in effector molecules [1]. These globular domain types include SH2s (phosphor-tyrosine readers), FHAs/14-3-3s/BRCTs (phosphor-serine/threonine readers), Bromo/YEATS domains (acetyl-lysine readers), UBA/UIM/GAT/CUE domains (ubiquitinated-lysine readers), Chromo/PHD/Tudor/BAH domains (methylated-lysine readers), and Tudors (methylated-arginine readers). In this review, we will focus on just one single thread in this hairball of signaling networks: the PRMT5–SND1 axis.

**Figure 1:**
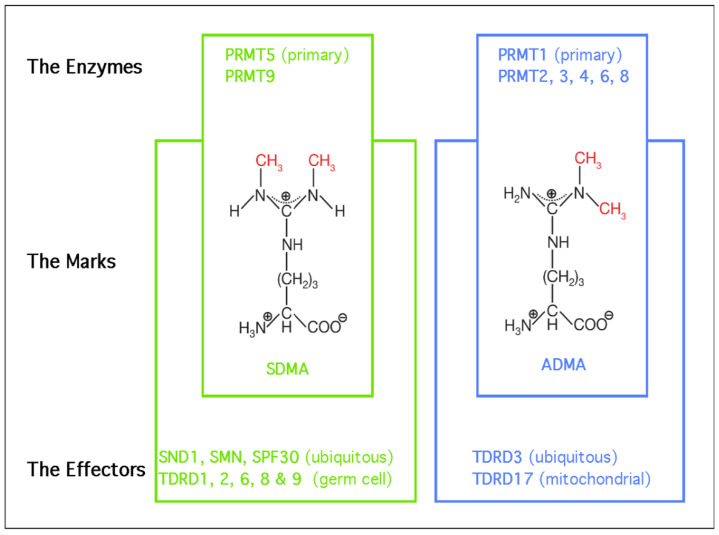
The PRMTs, the marks they deposit, and the effectors of those marks. PRMT5 and PRMT9 deposit the SDMA mark, in “green”. PRMT1-4, 6 and 8 all deposit the ADMA, in “blue”. Methyl groups are highlighted in “red”. The effectors, or readers, of the methyl marks are Tudor domain-containing proteins.

Arginine residues are frequently methylated post-translationally, and these modifications come in one of three flavors: monomethylarginine (MMA), asymmetric dimethylarginine (ADMA), or symmetric dimethylarginine (SDMA) (Figure 1). These methyl-mark additions occur at the peripheral omega nitrogen of arginine guanidine moieties and are commonly seen at glycine- and arginine-rich (GAR) motifs. Protein arginine methyltransferases (PRMTs) are the family of nine closely-related enzymes which deposit all three of these marks [2]. There is a tenth arginine methyltransferase called NDUFAF7, which is not a member of PRMT family, and seems to be a dedicated mitochondrial enzyme [3]. PRMT1 is the primary Type I PRMT, which deposits the majority of ADMA marks. Other Type I PRMTs include PRMT2, PRMT3, PRMT4/CARM1, PRMT6 and PRMT8. PRMT7 is capable of depositing only MMA marks, and it is referred to as a Type III enzyme. That leaves the Type II enzymes, which deposit SDMA marks. PRMT9 has just one known substrate (SAP145) [4], and NDUFAF7 is also dedicated to just one mitochondrial substrate (NDUFS2) [3]. All the remaining proteins marked with SDMA are thought to be PRMT5 substrates. Arginine methylation is a relatively abundant PTM and over the years, a number of studies have reported similar rations of the different types of arginine methylation in both tissue and cell lines [5,6,7], which generally breaks down to 1500:3:2:1 for Arginine:ADMA:SDMA:MMA. So, approximately 0.6% of all arginine residues found in proteins are arginine methylated.

Tudor domains are the only globular folds known to bind methylated arginine motifs [8]. This domain type was first identified in the *Drosophila melanogaster* Tudor protein which contains repeating domains that are present in a number of other proteins in many different species [9]. A detailed understanding of how Tudor domains interact with SDMA motifs was first gleaned from biochemistry and structure studies involving the human survival motor neuron (SMN) protein [10,11], which is mutated in spinal muscular atrophy syndrome. All in all, eight Tudor domain-containing proteins have been reported to be methyl-arginine readers. The vast majority of methyl-arginine readers recognize SDMA motifs, including SMN, SPF30, and SND1, which are ubiquitously expressed, and TDRD1, TDRD2, TDRD6, and TDRD9, which are all germ cell-specific proteins. TDRD3 is currently the only known ADMA motif effector protein that is also ubiquitously expressed (Figure 1).

**Figure 2:**
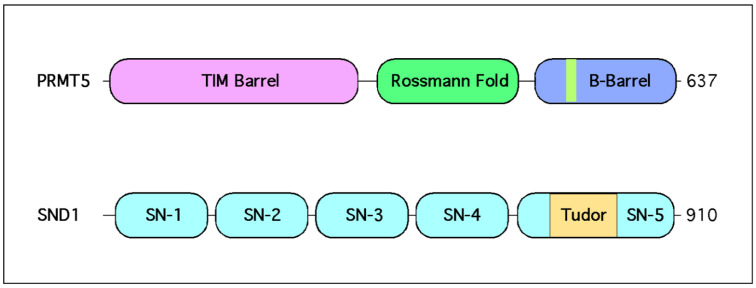
PRMT5 and SND1 structural domains. PRMT5 contains three distinct regions. A TIM barrel, which contains eight consecutive alpha helices, followed by a Rossmann fold, which contains the amino acid residues that bind S-adenosylmethionine. The C-terminus of PRMT5 contains a beta barrel in which the dimerization domain has been mapped. In hetero-octamerization complexing with MEP50, PRMT5 dimerizes head to toe with other PRMT5 proteins. SND1 contains four intact SN-like domains capable of binding nucleic acids and have nuclease activity. A fifth truncated SN-like domain is split by the Tudor.

PRMT5 has emerged as an important player in HCC [12], the fourth leading cause of cancer mortality in the world [13]. This link to HCC is strengthened by the fact that a downstream reader of the PRMT5-deposited SDMA marks—SND1—has been identified as a driver of HCC formation [14], though the precise molecular mechanism of action remains poorly understood. This review focuses on summarizing key biological functions of the PRMT5–SND1 reader–writer pair (Figure 2), then surveys what is known about these proteins as they relate to HCC, and concludes with speculation on unexplored avenues of therapeutic modulation of methylarginine levels in HCC as a potential form of treatment.

## 2. Biological Roles of PRMT5—The Primary Depositor of SDMA Marks

PRMT5 is the predominant Type II arginine methyltransferase, indicating that it confers both MMA and SDMA marks on target substrates in a distributive manner [15] (Figure 1). The primary targets of PRMT5 methylation are RNA-binding proteins, epigenetic modulators and core histones, which has implicated this enzyme in transcriptional regulation and the control of faithful alternative splicing [16]. PRMT5 is not enzymatically active on its own, and is found in a protein complex called the methylosome.

### 2.1. PRMT5 Forms a Stable Complex with MEP50

Regardless of the methylation target, PRMT5 requires the co-factor methylosome protein 50 (MEP50) for stability and enzymatic activity [17]. MEP50 is also referred to as WDR77. Loss of MEP50 results in the destabilization of the PRMT5 protein and vice versa [18]. Enzymatic activity is further dependent on hetero-octamerization of these two proteins to form a complex of four PRMT5 molecules and four MEP50 molecules [19,20,21]. MEP50 has also been identified as a potential coactivator of the androgen receptor [22], but it is unclear whether PRMT5 is recruited with MEP50 in this context, or whether it functions independently. Importantly, HeLa cell fractionation studies from a sucrose gradient indicate that PRMT5 and MEP50 only occur together and are not found in a complex without the other, nor do they exist in the free un-complexed form [17]. Similar fractionation experiments using *Xenopus* egg extracts and gel filtration also reveal the existence of a single PRMT5–MEP50 complex and no free monomeric form of either protein [23]. Thus, these two proteins are tightly complexed and likely do not function independently.

### 2.2. The Methylosome is Targeted to Distinct Substrates by Adaptor Proteins

The PRMT5–MEP50 protein complex requires additional adaptor proteins to aid in identifying substrates that will be targeted for symmetric methylation. There are five known adaptors that link the methylosome to its substrates, and these are pICln, RIOK1, COPR5, Sharpin and OXR1A. pICln is a spliceosome assembly chaperone, which recruits PRMT5 to facilitate the efficient methylation of SmB/B’ and SmD1/2 [24,25], as well as a number of ribosomal proteins [26]. A second adaptor is RIOK1, which is critical for the methylation of nucleolin by PRMT5–MEP50 [27], and is important for pre-rRNA transcription and processing. RIOK1 and pICln compete for binding, suggesting that there may be a common pocket for adaptor protein binding, either on PRMT5 or MEP50 [27]. COPR5 is the third adaptor to be identified, and it recruits PRMT5 activity to nucleosomes to support the deposition of H3R8me2s and H4R3me2s marks, in its role as an epigenetic regulator [28]. The fourth adaptor is Sharpin, and this interaction targets PRMT5 to methylate the transcription factor SKI [29]. Finally, OXR1A also regulates PRMT5′s ability to methylate histones, and it is the H3R2me2s methylation that is stimulated by this adaptor [30]. OXR1A and PRMT5 interact in the pituitary gland and regulate growth hormone expression, which in turn impacts liver metabolism. Both RIOK1 and pICln were identified in independent shRNA screens that also identified PRMT5 as a vulnerability in MTAP-null tumors [31,32,33], further supporting the key role that these adaptors play in helping the PRMT5–MEP50 methylosome find its targets for methylation.

### 2.3. The Identification of PRMT5 Substrates Implicate It in the Regulation of Transcription, Splicing, Signal Transduction and the Repair of DNA Damage

The initial characterization of PRMT5 as an arginine methyltransferase revealed that it methylates H2A and H4, using an in vitro methylation assay [34]. Importantly, the first five residues of H2A and H4 are identical (SGRGK…), and it is the arginine in position 3 that is methylated by PRMT5. Knockout studies showed that the H2AR3me2s modification is particularly sensitive to PRMT5 loss in vivo [35]. Sm proteins were also shown to be methylated by PRMT5 early on in the study of this PRMT [24]. Since then, over the last twenty years, a large number of PRMT5 substrates have been identified [16]. These studies have been spurred on by the development of efficient pan-substrate antibodies that recognize Rme2s marks on different substrates, and can be used to enrich for methylated peptides from tissue and cell extracts, which can then be identified by mass spectrometry. The first such substrate screens were performed by the Richard lab [36], and subsequent screening studies have dramatically expanded on the number of known symmetrically methylated proteins into the 100s [37]. Gene ontology (GO) analysis of the identified PRMT5 substrates reveals strong enrichment of RNA splicing and processing, as well as PTM regulated gene expression pathways and, to a lesser extent, translation.

### 2.4. PRMT5 Functional Misdirection Due to Cross-Reactivity with the FLAG Antibody

The PRMT5 field has been confounded by the occurrence of a major artifact of tandem affinity protein (TAP) complex purifications that use the FLAG-tag. Over the years, it is well established that when purifying a FLAG tagged protein using FLAG-M2 beads, a major contaminant is the PRMT5–MEP50 protein complex, because the M2 antibody binds directly to PRMT5 and purifies both it and its associated proteins. This was first reported by Danny Reinberg’s lab over 18 years ago [38]. Subsequent studies by the Siekhattar lab reported the same thing [39]. PRMT5 is also listed as a common contaminant of FLAG immunoprecipitation experiments [40]. Most recently, the CRAPome was published, which highlights the major problems with affinity purification-mass spectrometry data sets [41]. Indeed, they showed that 94% of all FLAG purifications data sets detect PRMT5 peptides. Thus, the misassignment of PRMT5 in many FLAG-tagged protein complex purifications has led many researchers astray, and these artifacts have found their way deep into the published literature.

### 2.5. Mouse Models Reveal a Number of Biological Roles for the Methylosome

It is very likely that loss of PRMT5 and MEP50 in mice will phenocopy each other, as they are codependent on each other for protein stability. Indeed, the interdependence and essentiality of MEP50 and PRMT5 complexing is supported by the fact that the mouse knockouts of both PRMT5 and MEP50 result in early lethal developmental defects. MEP50 knockout mice display an early embryonic lethal phenotype with no null embryos detected at E8.5 [42]. The PRMT5 knockout mice also display a very early embryonic lethal phenotype [35].

The early lethality of these total knockouts has made it necessary to generate conditional alleles for both PRMT5 and MEP50, to help elucidate the biological roles of this protein complex in vivo. Importantly, conditional knockouts of PRMT5 have provided additional insights into its roles in T and B cell development, limb development and neural development [43,44,45,46,47]. A conditional allele for the study of MEP50 loss in adult mice is also available, but has only been used in two studies related to prostate development [48] and lung development [49], although the conditional knockout was performed ex vivo in the latter study.

The first conditional PRMT5 knockout mice were generated by crossing PRMT5^fl/fl^ and Nestin^cre^ mice, which resulted in postnatal lethality in all homozygous null mice, and implicated PRMT5 in neuronal development [43]. Further exploration determined that this mortality was linked to splicing variations of Mdm4, which induces a p53 response, leading to severe cranial abnormalities. Subsequently, PRMT5 was conditionally knocked out in oligodendrocytes, using Olig1^cre^, and identified as a key factor for myelination [50]. Myelin basic protein has long been known to be a robust substrate for PRMT5 in in vitro studies, and the myelination defect in the conditional knockout mice provides in vivo evidence for the functional importance of this PTM [51].

A number of additional conditional knockouts of PRMT5 have been performed in adult mice. PRMT5 is also essential for the initiation and maintenance of hematopoiesis [44,52]. Methyl-transferase localization appears to impact the modification of splicing machinery, whereas loss of PRMT5 results in alternative splicing defects via intron retention and exon skipping, which is critical for hematopoietic stem cell quiescence and viability [44,53]. Both conditional knockout and small-molecule inhibitor studies reveal that loss of PRMT5 has anti-tumor activity against MLL-rearranged acute myeloid leukemia (AML) likely due to hypomethylation of essential splicing factor like SRSF1 [54,55], and further vulnerability of cancer to PRMT5 loss is bestowed on the tumors that harbor driver mutation in splicing factors [56]. Using a CD4^cre^, it was recently shown that PRMT5 is dispensable for late T cell development, and is required for peripheral T cell expansion and survival [46]. The removal of PRMT5 activity from pancreatic beta cells, using the Pax^cre^, reveals its role on regulation of insulin expression in vivo [57]. PRMT5 has also been shown to play a role in muscle stem cell expansion in adult mice (using Pax7^cre^), but does not seem important for the proliferation and differentiation of myogenic progenitor cells during embryonic development [58].

In a mouse embryo developmental biology setting, PRMT5 has been identified as a key for certain differentiated chondrocytes, and in this case Prx^cre^ was used to remove PRMT5 from developing limb buds [59]. Conditional deletion of PRMT5 in hind limbs of mice led to severe phenotypes of atrophied long bone and knee. While essential for some chondrocyte lineages, PRMT5 is dispensable for general chondrocyte maintenance in adult mice. Inactivation of PRMT5 in germ cells (using Tnap^cre^) results in defects in spermatogenesis [60], and loss of PRMT5 in the developing lung epithelial cells (using Shh^cre^) causes defects in branching morphogenesis [61].

Although PRMT5 biology has been studied extensively through conditional knockouts in both adult mice and embryos, far fewer mouse genetic studies have been performed with the other key component of the methylosome—namely MEP50. Importantly, a conditional allele for mouse MEP50 has been generated [48]. However, it has only been used in one study, and that was to investigate the role of MEP50 in the prostate (which we mentioned earlier). Using the Probasin^cre^ mouse, MEP50 was conditionally removed from all lobes of the developing mouse prostate. This inactivation of PRMT5 had a severe inhibitory effect on prostate development during embryogenesis, which is likely mediated by the deregulation of androgen receptor (AR) target genes due to the ability of MEP50 (and likely PRMT5) to function as an AR cofactor.

While PRMT5 and MEP50 knockouts have been shown to be essential for many key developmental pathways, PRMT5 also harbors many oncogenic characteristics through its ability to repress the expression of the tumor suppressors ST7 and NM23 [62]. Likewise, loss of E-cadherin, a characteristic of epithelial to mesenchymal transition (EMT) which is key for metastasis, is actively repressed through the binding of PRMT5 and AJUBA to SNAIL [63]. Overexpression of PRMT5 further induces hyperproliferation of cell lines in culture [64,65]. In addition, PRMT5 has been shown to be overexpressed in many different cancers including gastric [66], colorectal [67], lung [68,69], lymphoma [64], ovarian [70], melanoma [71], and glioblastoma [72,73]. The focus of this review is on the overexpression of PRMT5 in HCC and there are numerous reports of elevated PRMT5 levels in liver cancer [12,74,75,76,77,78]. Most of these published studies demonstrate that PRMT5 is overexpressed in many different cancer types, and PRMT5 overexpression correlates with aggressive tumors and poor prognosis. However, it is not clear whether PRMT5 is an oncogenic driver, or whether the elevated PRMT5 levels are an aftereffect of a transformed state. In other words, it is still unknown whether high PRMT5 expression is a cause or a consequence of cellular transformation.

## 3. Biological Roles of SND1—A Major Reader of SDMA Marks

SND1 (also known as TSN, p100, or TDRD11) is a ubiquitously expressed Tudor domain-containing protein [79]. Unique characteristics of SND1 include four tandem SN domains which convey nucleic acid binding and nuclease activity [79,80] (Figure 2). The SN domains are followed by a single Tudor domain that exclusively recognizes SDMA marks, which is fused to a fifth split SN domain [79,81]. This dual ability to simultaneously interface with nucleic and amino acids allows SND1 to impinge on a wide range of different signaling pathways. Some have claimed that this multifacility endows SND1 with the ability to “positively impact all hallmarks of cancer” [82]. Despite its preferential SDMA reading specificity, SND1 has been called a promiscuous binder given its affinity for RNA and DNA, and it regulates multiple pathways that control various aspects of gene expression [83,84].

### 3.1. The Tudor Domain of SND1 Interacts Selectively with SDMA Marks

Methylated lysine motifs are bound by at least eight different domain types—Chromo, PHD, MBT, Tudor, PWWP, Ank, BAH and WD40 domains. In the case of methylated arginine motifs, only members of the Tudor family are known effectors, with a handful of Tudor domain-containing proteins either binding SDMA or ADMA marks [8]. Importantly, there are a few individual PHD and WD40 domains whose binding affinity is also impacted by arginine methylation. Tudor domains were identified simultaneously by two research groups, which both realized that the *Drosophila melanogaster* Tudor protein contains previously unrecognized repeating domains, which were found in a number of other proteins in many different species [9,85]. Interestingly, SND1 was one of the first proteins to be identified as a Tudor domain-containing protein [85]. Initial structural studies involving the Tudor domain of SND1 identified an aromatic cage that suggested it might recognize methylated peptide ligands [86]. Subsequent crystal and NMR structural studies found that the extended Tudor domain of fly SND1 bound a short GAR motif from SmB, only when this motif was symmetrically dimethylated [87]. Finally, work involving the human SND1 protein revealed that it bound PIWIL1 in an arginine methylation-dependent manner, with a strong preference for SDMA motifs over ADMA motifs [88]. Thus, SND1 reads marks that PRMT5 deposits.

### 3.2. SND1 as a Transcriptional Coactivator

SND1 was originally identified as a transcriptional coactivator of EBNA2 (Epstein–Barr nuclear antigen-2) [89], which interacts with many general transcription factors and coactivators, and positions SND1 as a central player in transcriptional regulation. Indeed, SND1 has been found to directly engage with a number of transcription factors. Signal transducer and activator of transcription 6 (STAT6) is a key player in transcriptional activation upon IL-4 stimulation. SND1 acts as a transcriptional coactivator by binding the *C*-terminus of STAT6 along with RNA Polymerase II. In this way, SND1 acts as a bridge between STAT6 nuclear localization and transcriptional activation [90]. A second STAT protein, STAT5, induces transcriptional activation in response to lactogenic hormones, which is facilitated in a similar fashion by SND1 binding the *C*-terminal transcriptional activation domain [91]. SND1 also functions as a coactivator for the c-Myb transcription factor [92], and in this context it is regulated by phosphorylation. Using a protein domain microarray approach, we identified the Tudor domain of SND1 as a reader of a PRMT5 deposited SDMA motif within the E2F1 transcription factor [81]. E2F proteins are widely known for their central role in transcriptional activity and their close association to proliferation and cancer. Follow-up mechanistic studies by the La Thangue group revealed that the recruitment of SND1 to arginine methylated E2F1, results in cross-talk between transcriptional regulation and altered splicing regulation of a subset of E2F1 transcriptional target genes [93]. Independent luciferase-based assays have validated the ability of SND1 to coactivate E2F1 transcriptional activity [94]. SND1 is also an interactor and coactivator of the transcription factor peroxisome proliferator-activated receptor γ (PPARγ), and regulates adipogenesis [95]. Thus, SND1 interacts directly with a number of transcription factors (STAT5/6, c-Myb, E2F1 and PPARγ) and promotes their transcriptional activity.

### 3.3. SND1 Is a Splicing Factor

The splicing of precursor mRNA is a highly ordered process that is orchestrated by the spliceosome. The spliceosome is composed of five small nuclear ribonucleoprotein particles, which by definition harbor a mix of small nuclear RNAs (snRNAs U1, 2, 4/6 and 5) and proteins (Sm proteins plus additional splicing factors). SND1 interacts with both the RNA and protein components of the spliceosome. Indeed, due to its unique structure, with a Tudor domain for protein binding and SN domains for RNA binding, it plays a role in spliceosome complex formation. The Tudor domain of SND1 interacts directly with the arginine methylated forms of SmB/B’ and SmD1/D3 [96], and also the splicing factor Sam68 [97]. The interaction of SND1 with the U RNAs is likely mediated through the Sm core proteins, which bind directly to U1, U2, U4, U5, and U6 snRNAs, as the Tudor domain of SND1 can pulldown U RNAs, but the SN domains cannot [98].

As mentioned above, SND1 directly associates with a number of transcription factors. It has been proposed that the recruitment of SND1 by transcription factors to enhancer/promoter elements can directly impact the alternative splicing of the transcripts that are being activated by that particular transcription factor, at least in the case of E2F1 [93]. In summary, PRMT5 is a key regulator of RNA splicing [55], and as an effector molecule for SDMA marks deposited by PRMT5, it is not surprising that SND1 is also integral to the maintenance of normal splicing programs that can go awry in a cancer setting.

### 3.4. SND1 Regulates RNA Stability

Apart from splicing, SND1 is involved in many other aspects of RNA biogenesis. SND1 is not only a component of the spliceosome, but also the RNA-induced silencing complex (RISC), which are both ribonucleoprotein particles. The RISC is a “carrier” of miRNA and siRNA, which when loaded with Argonaute can target mRNA for cleavage to regulate gene expression. A biochemical purification of RISC in *Drosophila* identified SND1 along with Argonaute 2 and VIG-1 [99], and SND1 is enriched in size fractioned extracts that also contain the 250 kDa miRNA complex. SND1 was also confirmed to be a component of the mammalian RISC enzyme. SND1 was shown to have ribonuclease activity that is specific for inosine-containing dsRNAs [100], and it was subsequently found that SND1 selectively degrades a highly edited pri-miR-142 that is not processed by Drosha [101]. SND1 has four intact SN domains (the 5th SSN domain is split by the Tudor domain), and structural studies have revealed that a minimum of two tandem SN domains are necessary and sufficient for RNA binding [102]. Recent studies have found that SND1 is involved in regulating the turnover of a sub-set of mature miRNAs with a common CA/UA dinucleotide sequence signature [103]. This process is known as Tudor-staphylococcal/micrococcal-like nuclease (TSN)-mediated miRNA decay (TumiD), and it is promoted by the UPF1 helicase [104]. Thus, as a component of RISC, SND1 is actively involved in processing miRNAs.

### 3.5. SND1 as a Component of Exosome Cargo

Extracellular vesicles (EVs), including exosomes and microvesicles, carry high levels of SND1 as part of their secreted cargo. Exosomes are critical carriers of molecular and signaling information in the extracellular environment of organ systems, where they transfer molecules from one cell to another via membrane vesicle trafficking. Exosomes are approximately 100 nm in size, and are produced in the endosomal compartment (the Golgi network) of most eukaryotic cells. With the development of cancer, exosomes become important messenger packages that “speak to” the tumor microenvironment. The first hint that SND1 was sorted into vesicles came from a study showing the presence of SND1 protein in lipid droplets that are generated by milk secreting cells [105]. Subsequent work has revealed that EVs are enriched for miRNAs, mRNAs and Ago2, which a key protein component of RISC [106,107,108]. SND1 is also an integral component of RISC [99], and it is thus not surprising that it is also part of these miRNA/Ago-enriched EVs. An analysis of the changes in the protein composition of exosomes, after ionizing radiation, reveals an increase in SND1 [109]. In addition, patient urinary EVs, which are secreted by bladder cancer cells also contain high levels of SND1 [110]. Exosome-mediated intracellular communication within the tumor microenvironment seems to play an important role in the development and progression of HCC [111].

### 3.6. SND1 Expression Patterns at the RNA and Protein Levels

**Figure 3:**
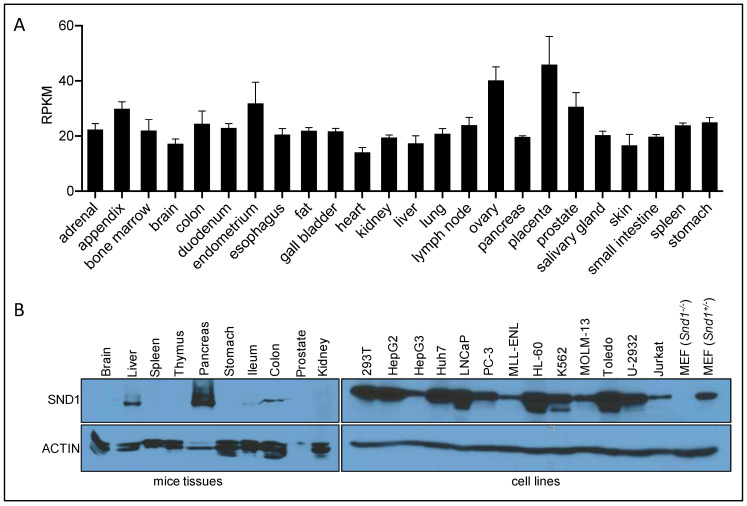
SND1 RNA and protein expression. (**A**) SND1 RNA expression in different human tissues. Data was obtained from PRJEB4337 (https://www.ncbi.nlm.nih.gov/bioproject/PRJEB4337/). The expression values of SND1 in 24 human tissues were obtained from RNA-seq RPKM (Reads Per Kilobase per Million mapped reads) values and analyzed by Graphpad. (**B**) SND1 protein expression in different cell lines and mice tissues. MEF were generated from E13.5 mouse embryo following a standard 3T3-MEF generation protocol. MEF cells and other cell lines were lysed. Total cell lysates were analyzed by Western blotting. Different tissues from 8-week-old FVB mice were homogenized and lysed, total tissue lysates were analyzed by Western blotting. The antibodies used were anti-SND1 (Bethyl, #A302-883A) and anti-ACTIN (Sigma, #A1978).

Detailed profiling of SND1 expression has found it to be ubiquitously expressed, with the highest expression in proliferating cells and active secretory organs such as exocrine pancreas, lactating mammary glands and the liver [112]. Western analysis of SND1 reveals that it is most highly expressed in the pancreas and the liver [88] (see Figure S1 in Liu et. al., 2010). We have reproduced this data and also see a very similar expression pattern (Figure 3). RNA-seq analysis, curated by the NCBI, was performed on 24 different human tissues from 95 individuals, and also reveals fairly ubiquitous RNA expression of SND1, with approximately a two-fold variable between tissues (Figure 3A). We performed Western analysis on protein extracted from a panel of cell lines, and we observed ubiquitous expression of SND1, albeit at varying levels (Figure 3B). When comparing the protein and RNA expression of SND1, there seems to be a disconnect between the high protein levels of SND1 in pancreas and the liver, and the equal and ubiquitous RNA expression of SND1 in different human tissues. This observation could be explained by the post-transcriptional regulation of SND1 possibly by the proteasome. Indeed, mass spectrometric analysis of SND1 reveals at least 10 different lysine residues that can be ubiquitinated (see the CST—PhosphoSite database). However, no studies have yet been performed to evaluate the protein stability of SND1 in different tissue settings. Alternatively, certain organs such as the liver and pancreas harbor levels of exosome activity, and SND1 may by sorted and secreted in the tissues from these organs, resulting in extracellular accumulation and retention.

### 3.7. Mouse Models of SND1 Overexpression Support Its Potential Oncogenic Functions

The SND1 knockout mouse has yet to be described. However, it does seem that this mouse has been generated [95], but the knockout phenotype was never presented, and these mice were only ever used to generate SND1 knockout mouse embryonic fibroblasts (MEFs) for further analysis [94,113]. Phenotyping performed by the mouse genome informatics (MIP) project at the Jackson Labs and the international mouse phenotyping consortium (IMPC), which both perform high-throughput phenotyping of spontaneous and trapping mutant mice, suggests that the SND1 knockout is partially viable, with knockout mice appearing at lower than expected Mendelian rations (approximately 50% of the expected ration). This data suggests that adult SND1 knockout mice will be available for detailed analysis (albeit at low numbers). However, very little information has yet been gleaned from the systemic knockout of SND1. It would be very informative to compare an SND1 knockout phenotype to a SND1 Tudor-dead knockin phenotype, as this would reveal the importance of the methylarginine reader abilities of SND1, and reveal what signaling pathways are dependent of SND1’s ability to read PRMT5 deposited marks, and what SND1 functions are independent of PRMT5 activity.

An overexpression model has revealed that SND1 is a driver for HCC when the induction of expression is focused on the liver, using an Albumin promoter (see Section 4.2) [14]. Similar overexpression models for PRMT5 would be extremely valuable to investigate whether this mouse would phenocopy the SND1 as a driver of HCC. Although we have generated other PRMT overexpression transgenic mouse models (including PRMT1, CARM1 and PRMT6) [114], the PRMT5 overexpression transgenic mouse has not yet been developed.

### 3.8. Mouse Syngeneic Tumor Models Reveal a Role for SND1 (and PRMT5) in Antitumor Immunity

The melanoma B16F10 cell line has been used to investigate the role of SND1 in facilitating immune evasion of tumor cells [115]. B16F10-SND1-KO cells were transferred into the flank of syngeneic mice and monitored over a number of days. The resulting tumor size and weight were smaller for growths seeded with SND1-KO cells than in the control parental cell. Furthermore, it was found that SND1-null tumors elicited a robust immune response, when compared to the parental cells. This suggests that the loss of SND1 may sensitize tumors to immune checkpoint inhibitors. Importantly, very similar experiments involving siRNA-mediated PRMT5 knockdown or PRMT5 inhibition by small-molecule treatment in B16F10 cells also showed that the presence of PRMT5 activity attenuates immune checkpoint therapy [116]. Thus, the loss of either PRMT5 or SND1 will convert an immunologically “cold” microenvironment into a “hot” one, further supporting a mechanistic link between this writer–reader pair. HCC may be sensitized to respond to immune checkpoint inhibitors (which block PD1, PD-L1 and CTLA-4 activity) by prior treatment with PRMT5 inhibitors.

### 3.9. SND1 Is Likely an Oncogene

Like PRMT5 [16], SND1 is upregulated in many different cancer types [117,118]. There is a particular interest in HCC, stemming from the observation made ten years ago, that SND1 protein levels are elevated in HCC and that its expression increases with the stage of the disease [119]. Interestingly, the analysis of RNA expression databases (TCGA and GEO) does not support a role for SND1 expression in the clinical progression of liver cancer [117], but the analysis of protein expression by immunohistochemistry does [119]. This suggests that SND1 may be post-transcriptionally regulated in certain tissues, as we have eluded to above (Figure 3). We will next summarize the reported roles of PRMT5 and SND1 in HCC, which can serve as a pre-clinical (mouse) and clinical (human) model system for understanding the link between this enzyme and its effector.

## 4. Hallmarks of HCC

Liver cancer comes in a variety of types and frequencies, from the common HCC and cholangiocarcinoma to the rare liver angiosarcoma and pediatric hepatoblastoma. HCC is cited as constituting approximately 75% of all liver cancers according to the Cancer Treatment Centers of America. Among all cancers, HCC is the fourth leading cause of cancer mortality worldwide [13]. Traditionally, HCC has affected more males than females, though recent studies have begun to suggest occurrence may have less gender disparities than previously thought [120,121]. Induction of HCC is often preceded by other hepatic ailments which ultimately develop into HCC. Non-alcoholic fatty liver disease (NAFLD) is one such malady that ultimately leads to HCC and is associated with obesity and metabolic syndrome [122,123]. Progression towards HCC from NAFLD often occurs in successive stages from NAFLD which develops into non-alcoholic steatohepatitis (NASH), increasing to fibrosis and cirrhosis, concluding with HCC and metastasis [124].

Atypical lipid accumulation is characteristic of hepatic damage and malignancies and HCC is no exception [125,126]. Hallmark metabolic dysregulation in hepatic tissues include increased de novo synthesis of lipids over extracellular lipid uptake to fulfill the lipid requirements needed for excessive cell division in the transformed state [126,127,128]. Upswing in lipogenesis arises in part from the liver being the center of lipid synthesis allowing a microenvironment permissible for de novo lipid synthesis. Sterol regulatory element-binding protein (SREBP) is a transcription factor directly upregulated in hepatocytes with active de novo lipogenesis. Accordingly, in HCC and premalignant hepatocytes, lipid accumulation is a tell-tale indicator of increased HCC risk. Lipid accumulation can be grossly visualized as lipid droplets within cells. Additional markers of HCC used in the clinic include measurement of alpha-fetoprotein (AFP), Alanine transaminase (ALT), and Aspartate transaminase (AST). Each of these enzymes, when elevated in the blood stream, can suggest malignancy and dysregulation of hepatocytes as they are typically seen in low abundance extra-hepatically. HCC is highly vascularized allowing excessive signaling and growth factor secretion for rapid expansion of cancer cells and is subsequently supported by sustained nutrient availability [129]. Hepatitis B/C (HBV and HCV, respectively) is the most common risk factor for developing HCC. Many reviews have explored HBV and HBC as they relate to HCC. Interestingly, PRMT5 can methylate the HBV core protein [130], and regulates it nuclear accumulation. Thus, there may be cross-talk between PRMT5–SND1 axis and hepatitis, but this issue will not be further addressed here.

### 4.1. PRMT5 and HCC

PRMT5 has, in recent years, become an increasingly prominent character in HCC research. Multiple studies have reported a worse prognosis of HCC patients with increased PRMT5 expression [12,74,75,76,131]. PRMT5 combined with the lysine methyltransferase SET8 have been identified as predictors of overall survival and recurrence in HCC patients [131].

EMT and invasion are key hallmarks of metastasis and PRMT5 has been identified as important in both. In vitro knockdown of PRMT5 in HCC and colon cancer cells decreases matrix metalloproteinase-2 expression [75]. This impaired expression decreases invasiveness of these lines along with decreasing proliferation which is supported by another study confirming increased HCC proliferation in PRMT5 competent lines [78]. A current model of E-cadherin depletion, characteristic of EMT, proposes the zinc finger domains of SNAIL directly bind the E-cadherin enhancer. AJUBA links PRMT5–MEP50 complex to SNAIL thereby permitting PRMT5 to mediate the SNAIL-dependent gene repression of E-cadherin [63].

A cellular defense against EMT in hepatocytes is the transcription of hepatocyte nuclear factor 4α (HNF4α). HNF4α has been shown to re-differentiate HCC cells towards hepatocytes and repress EMT, thereby blocking hepatocarcinogenesis [121,132,133]. While HNF4α works to drive differentiation towards hepatic cellularity, PRMT5 antagonizes HNF4α expression assisting in liver cancer stem cell maintenance. In HCC cells, PRMT5 binds H4R3 generating H4R3me2s at the HNF4α promoter. This methylation of H4R3 represses HNF4α transcription, while inhibition of PRMT5 activity restores HNF4α transcription and differentiation activity [78].

Another tumor suppressor, BTG2, is known to suppress proliferation, and is typically inhibited in cancers. While tumor suppressors are commonly inactivated by genetic mutations, deleterious mutations in BTG2 have not to date been identified, suggesting its downregulation may be a result of epigenetic reprogramming. PRMT5 has recently been linked to repressing BTG2 expression (again, in the context of HCC) through ERK signaling, though the mechanism of repression remains unknown [74].

As previously mentioned, lipid accumulation is concurrent with hepatic damage [125]. Lipid accumulation is driven by de novo lipogenesis over extracellular uptake, implying that drivers of lipid synthesis harbor oncogenic potential in hepatocytes. Sterol regulatory element-binding protein 1 (SREBP1) is a transcription factor that regulates the expression of genes involved in the synthesis of fatty acids, triglycerides and phospholipids, and has recently been shown to directly interact with PRMT5 [134]. Furthermore, PRMT5 methylates SREBP1, which stabilizes this transcription factor and helps promote the expression of its target genes and consequently also de novo lipogenesis. The overexpression of PRMT5 in HepG2 cells increases the levels of intracellular triglyceride levels, and conversely, the knockdown of PRMT5 results in a decrease in Oil red-O staining (a marker for intracellular lipid droplet accumulation). Furthermore, the overexpression of SREBP1 causes an increase in Oil red-O staining, which is not observed when the mutant form of SREBP1 (that cannot be methylated by PRMT5) is overexpressed. Thus, PRMT5 promotes de novo lipogenesis by methylating a single site on SREBP1 [134].

Long non-coding RNAs (lncRNAs) have emerged as key regulators of normal physiology as well as pathogenesis. Long intergenic non-coding RNA 1q21.2 (LINC01138) has been shown to correlate with PRMT5 expression as well as HCC tumor size, AFP levels, and hepatitis B surface antigen levels. PRMT5 and LINC01138 were shown to interact in HCC, which allowed PRMT5 to evade proteasomal degradation [77]. Increased PRMT5 stability may explain why we see increased protein expression of PRMT5, but not an increase in PRMT5 mRNA levels in some patient samples.

Recent findings reveal that PRMT5, in combination with SND1, promotes the dynamic regulation of E2F1 target genes. PRMT5 has been shown to promote cell growth, which contrasts to PRMT1 asymmetric dimethylation of E2F1 which promotes apoptosis [67,81]. PRMT5 methylation of E2F1, and the subsequent recognition of this methylated site by SND1, expands traditional E2F1 transcriptional control of genes to an extended set of targets that are traditionally poorly regulated by E2F1. Extended targeting is accomplished by E2F1-dependent alternative splicing of targets. This alternative splicing activity is dependent on both PRMT5 activity as well as SND1 recognition of SDMA marks on E2F1 [93]. While PRMT5 driven proliferation via E2F1 methylation has been identified, it remains unclear whether this signaling pathway is active or important for the development of HCC. However, given the emerging role of E2F1 in HCC [135], the activation of this regulatory node could be a critical consequence of elevated PRMT5 and/or SND1 protein levels.

### 4.2. SND1 and HCC

While the role that PRMT5 plays in the development of HCC is largely circumstantial—elevated PRMT5 levels clearly correlate with the promotion of HCC and poor prognosis of these cancer patients—its effector molecule, SND1, is more solidly implicated as a driver of HCC. Indeed, a landmark study of SND1 in HCC emerged from a mouse model with SND1 overexpression [14]. Transgenic mice carrying SND1 under the control of an albumin promoter/enhancer element, selectively overexpress SND1 in the liver, and this is sufficient to drive spontaneous HCC formation with partial penetrance. While half of the overexpressing mice develop HCC spontaneously, all overexpressing mice showed more aggressive tumors in HCC that is chemically induced by diethylnitrosamine (DEN). Hepatocytes from SND1 overexpressing mice have higher levels of spheroid-generating tumor-initiating cells. Furthermore, SND1 overexpression resulted in a steady proinflammatory state [14], similar to what is seen in chronic inflammation, a central hallmark of HCC progression.

SND1 contributes to alterations in the signaling cascades within HCC that control both transcriptional and post-transcriptional regulation. Angiotensin 2 receptor 1 (AT1R) mRNA stability is augmented through overexpression of SND1 [136]. Importantly, upregulation of AT1R is associated with both the progression of HCC, as well as unfavorable outcomes with respect to overall survival of cancer patients [137]. This increased stability of AT1R mRNA, and subsequent elevation of its protein levels, activates the ERK and SMAD signaling pathways, leading to a downstream increase in TGFβ signaling in HCC cells [138]. TGFβ signaling is known to drive proliferation and EMT progression [139]. Furthermore, TGFβ signaling also induces SND1 transcriptional activation in a feed forward loop [140]. This feed forward activity can be seen in other SND1-regulated pathways including NF-κB and SREBPs [141]. SND1 further promotes proliferative signals by degradation of miRNA via its nuclease domains. Elbarbary et. al. used transcriptome profiling to identify miRNAs that increase after knockdown or knockout of SND1, and show that these upregulated miRNAs in turn downregulate a cohort of mRNAs that are needed for G1/S transition [103]. We can speculate that the opposite is also true, when SND1 is upregulated, the G1/S transition may be shortened, and this would help explain one of the characteristics of liver cancer, which is a deregulated cell cycle [142].

NF-κB is a transcription factor that regulates innate immunity, and activation of this pathway promotes an inflammatory response and cellular growth. Chronic inflammation has been linked to many cancers, including HCC [143,144,145]. Indeed, chronic inflammation and hepatic injury serve as malignant drivers and precede 90% of HCC occurrences [146]. SND1 interacts with AEG-1 (also called metadherin) [119], which regulates multiple signaling pathways including NF-κB, PI3K/Akt and Wnt [147]. The activation of the NF-κB pathway by SND1 overexpression is reported to increase oncogenic miRNAs (oncomiRs) such as miR-221, that target and degrade tumor suppressor RNAs [80,148]. SND1 overexpression functions to increase inflammatory driving cytokines to promote HCC formation, as well as factors such as CXCL16 and angiogenin which promote angiogenesis. The inhibition of NF-κB blocks SND1-induced angiogenesis [80]. The benefits, to liver cancer cells, of having elevated SND1 can thus be explained, in part, by its pro-inflammatory and pro-angiogenic roles.

In order for cancer cells to persist and multiply, they evolve aggressive survival responses to stress signals to evade cell death pathways, which represents one of the major hallmarks of cancer. Stress granules are membraneless organelles that collect in the cytoplasm of cells, and serve as a means of stalling cellular machinery while the cell responds to the strain [149]. This suspended state can buy time sufficient for cells to respond with survival signaling, thereby evading apoptosis. SND1 has been shown to be enriched in stress granules induced from oxidative stress [150,151,152]. Whether SND1′s role is primarily as a nuclease or as a recruiting/scaffolding protein remains unclear. It has been noted, however, that phosphorylation of SND1 promotes its binding to G3BP [152], which in turn stimulates stress granule formation, suggesting that SND1 works in a recruiting role in stress granule formation. The ability of SND1 to help liver cancer cells evade cell death is not limited to its role in stress granule formation and function. SND1 can also promote the expression of UCA1 expression in HepG2 and SMMC-7721 cells [153]. UCA1 is an oncogenic lncRNA that has anti-apoptotic activity, and is itself a predictor of poor overall survival for patients with HCC [154]. Thus, high levels of SND1 helps tumor cells evade cell death.

A third survival pathway for cancer cells is DNA damage response. Using a laser microirradiation approach, SND1 is clearly localized to the laser-induced DNA damaged stripes [113]. Mechanistically, SND1 is recruited by PARP1 to damaged DNA, where it serves as a scaffold for chromatin remodeling proteins that then help facilitate DNA repair, such as SMARCA5 and GCN5 [113]. These two proteins are an ATP-dependent remodeler and a histone acetyltransferase, respectively, and they both act at sites of DNA damage to help open up chromatin so that the repair machinery can access the damaged DNA. SND1 overexpression thereby potentially provides a survival advantage in DNA damaged cells [82]. The combination of the liver as the body’s detoxification center, along with the ability of elevated SND1 levels to promote survival advantages under DNA damaging conditions, may partially explain why HCC is often chemoresistant and radioresistant. Targeting SND1 may reverse this resistance.

Like PRMT5, SND1 has been noted to have a variety of functions in lipogenesis. SND1 facilitates lipid droplet formation in mammary cells and hepatocytes. This association is lost in milk globules suggesting that SND1 association is specific to formation, but not maintenance of fat droplets [105,155]. SND1 overexpressing cells show a significantly altered lipoprotein secretion content and are saturated with phospholipids over other metabolically common lipids [156]. SREBPs appear to be regulated by SND1, although the difference in activity between normal and diseased states remains unknown [156]. More recently, it was found that hepatoma cells overexpressing SND1 display low triglyceride synthesis and accelerated cholesterol ester synthesis, likely because fatty acids are preferentially used for cholesterol esterification [157]. While profiling the target genes of SND1s transcriptional coactivator activity, using human hepatoma HepG2 cells, it was found that cohort of glycerolipid genes (such as *CHPT1*, *LPGAT1*, *PTDSS1* and *LPIN1*) are regulated by SND1, in response to proinflammatory TNFα signaling [83]. SND1 is thus key for sustaining glycerophospholipid homeostasis in human HCC cells. The roles of SND1 in lipid metabolism has recently been reviewed in detail [158].

## 5. Targeting Elevated SND1 Levels with PRMT5 Inhibitors

As highlighted above, PRMT5 is overexpressed in a large number of different tumor types, and the inhibition of PRMT5 has been linked to tumor regression in mouse models [2,16,54]. These findings indicate that PRMT5 might be a promising therapeutic target for both solid and liquid tumors. Indeed, PRMT5 is currently a very popular target for the development of small-molecule inhibitors by both pharma and biomedical startup companies. A search of the published and patent literature reveals the development of at least 13 distinct PRMT5 inhibitors (Table 1). These inhibitors have six different mechanisms of action (MOA) [159,160], namely (1) inhibitors that compete with SAM (but not the peptide substrate); (2) inhibitors that compete with peptide substrate (but not SAM-competitive); (3) inhibitors that block both SAM and the substrate peptide from binding; (4) covalent inhibitors that form a stable bond with Cys449 in the active site and prevent SAM binding; (5) the development of a PROTAC probe that is based on the GSK3326595 compound, and targets PRMT5 for proteasomal degradation; (6) an allosteric inhibitor which causes the formation of an 11 amino acid acidic loop that blocks both SAM binding and peptide substrate binding.

In addition to the panel of 13 different PRMT5 inhibitors that have been developed, it may be possible to target elevated PRMT5 levels using inhibitors for PRMT1 and perhaps even CARM1 (PRMT4). This is because there is clear evidence of redundancy between different PRMT family members. Indeed, we have shown that PRMT1 and PRMT5 share many substrates [7]. Furthermore, GSK and the Guccione lab have shown that PRMT5 and PRMT1 inhibitors function synergistically to target MTAP-null cancer cells [161] and tumors that are driven by splicing mutations [56]. Finally, using a CRISPR-screening approach, we have found that in the presence of PRMT5 inhibitors cells are sensitized to PRMT1 and CARM1 loss [162]. Thus, it may be important to evaluate the effects of a CARM1 inhibitor (GSK3359088) [163] and a Type I PRMT inhibitor (GSK3368715) [161] for their ability to retard the growth of tumors with elevated PRMT5–SND1 signaling.

## 6. Conclusion and Future Direction

HCC is a major health concern worldwide. It has a 20% five-year survival rate and 1% of the global population are expected to develop HCC in their life time. This disease poses a significant health burden which urges additional study. The majority of therapeutic options are hepatic resection and transplant, though transplant needs far outweigh available organs [13]. Accordingly, a more comprehensive molecular understanding of HCC development is needed to approach the HCC epidemic. The PRMT5–SND1 axis has emerged recently as a key point of inquiry, though we are far from understanding its intricacies in HCC. A lot of the interest in PRMT5 is driven by the fact that there are now very good inhibitors available that target this enzyme, raising therapeutic hope for diseases that are driven by PRMT5 overexpression, or by increased effector molecule activity (such as SND1 overexpression). SND1 has been shown to be a potent hepatic-oncogene, though many important questions still need to be addressed. Clearly, there remains a lot of low-hanging fruit to be picked; for example: (1) In the context of HHC, is SND1 the primary effector molecule for SDMA marks that are deposited by PRMT5? (2) Does SND1 compete with other Tudor domain-containing proteins such as SMN for binding to PRMT5 deposited marks? (3) Will PRMT5 inhibitors block the oncogenic effects of SND1 overexpression in the liver? (4) How important is the Tudor domain of SND1 for its oncogenic function? Many of these questions will require the development of new genetically engineered mouse models that will facilitate pre-clinical studies. A detailed mechanistic elucidation of the PRMT5–SND1 axis in HCC promises to illuminate novel and rational approaches towards treating this terrible disease.

## Figures and Tables

**Table 1:** PRMT5 inhibitor and their different mechanisms of action.

Company	Compound	Trials	MOA	Ref. or Patent
GSK/Epizyme	GSK3326595	Ph 1 NCT02783300Ph 2 NCT03614728	b	[164]
Pfizer	PF-06939999	Ph 1 NCT03854227	a	[165]
Janssen	JNJ64619178	Ph 1 NCT03573310	c	AACR
Prelude	PRT543	Ph 1 NCT03886831	a	[166]
Prelude	PRT811	Ph 1 NCT04089449	a	[166]
Prelude	C449	Pre-clinical	f	[167]
Eli Lilly	LLY-283	Pre-clinical	a	[168]
Argonaut	T1-44	Pre-clinical	b	[169]
Jian Jin	MS4322	Pre-clinical	d	[159]
Merck	Comp 1A	Pre-clinical	e	[160]
Aurigene	AU-14755	Pre-clinical	b	WO2019-180628
Angex	Not named	Pre-clinical	a	WO2019-112719
Jubilant	JPRMT5i	Pre-clinical	b	WO2019-102494

Mechanism of action (MOA): (a) SAM competitive; (b) SAM cooperative and peptide substrate competitive; (c) SAM and peptide substrate competitive; (d) PROTAC degrader; (e) allosteric modulator; and (f) covalent inhibitor. The “WO2019” numbers refer to patent submissions.
